# Radiogrametric Analysis of the Thoracic Limb Phalanges in Arabian Horses and Thoroughbred Horses

**DOI:** 10.3390/ani11082205

**Published:** 2021-07-26

**Authors:** Ozan Gündemir, Tomasz Szara, Gülsün Pazvant, Dilek Olğun Erdikmen, Sokol Duro, William Perez

**Affiliations:** 1 Department of Anatomy, Faculty of Veterinary Medicine, Istanbul University-Cerrahpasa, Istanbul 34500, Turkey; ozan.gundemir@istanbul.edu.tr (O.G.); gulsun@iuc.edu.tr (G.P.); 2 Department of Morphological Sciences, Institute of Veterinary Medicine, Warsaw University of Life Sciences—SGGW, 02-776 Warsaw, Poland; 3 Department of Surgery, Faculty of Veterinary Medicine, Istanbul University-Cerrahpasa, Istanbul 34500, Turkey; dilekolg@istanbul.edu.tr; 4 Faculty of Veterinary Medicine, Agricultural University of Tirana, 1029 Tirana, Albania; durosokol@ubt.edu.al; 5 Unidad de Anatomía, Facultad de Veterinaria, Universidad de la República, Montevideo 11600, Uruguay; vetanat@gmail.com

**Keywords:** horse, phalanx, radiogrametric study, veterinary anatomy

## Abstract

**Simple Summary:**

The aim of the research was to determine the radiogrametric features differentiating the phalanges of the thoracic limbs of Arabian horses and Thoroughbred horses including sexual dimorphism. Nine traits and three indexes were analyzed. Radiological measurements of phalanges showed that sexual dimorphism is not clearly marked and differences between breeds manifest themself mainly in the proximal phalanx measurements. None of the parameters tested in Thoroughbred horses differed significantly between males and females. The discriminant analysis enabled the correct classification of 89.33% of the proximal phalanx samples to the exact breed. This percentage was 77.33% in the case of the middle phalanx and 54.67% for the distal phalanx, respectively. The data obtained from this study can be used as a reference material for the radiogrametric evaluation of the skeleton of the manus in horses.

**Abstract:**

In this study, it was aimed to determine the statistical differences between Arabian horses and Thoroughbred horses based on X-ray images of forelimb digital bones. Latero-medial X-ray images of digital bones of thoracic limbs were taken of 25 Arabian horses and 50 Thoroughbred healthy horses. The difference between males and females within the breed was statistically analyzed as well. Nine measurements and three indexes taken from phalanges of thoracic limbs were used. Thoroughbred horses did not differ significantly between sexes, as indicated by the ANOVA. For the Arabian horses, the length of the middle of the proximal phalanx (*p* < 0.05), the length of the middle of the middle phalanx (*p* < 0.001), and the length of the dorsal surface of the distal phalanx (*p* < 0.05) measurement points were found to be differentiated between sexes. In the analysis made between Thoroughbred horses and Arabian horses with no respect to sex, the critical measurement was the depth of the caput of the proximal phalanx. The discriminant analysis enabled the correct classification of 89.33% of the proximal phalanx samples to the exact breed. The correct classification rate was 77.33% in the case of middle phalanx and 54.67% in the case of distal phalanx. Measurement results of the distal phalanx were found to be insignificant between both breeds and sexes. The radiological measurements of digital bones showed that sexual dimorphism was not too expressed and that decisive differences were found between the breeds.

## 1. Introduction

Radiographic imaging techniques have become more readily available, and are now widely used in veterinary environments. Beside clinical use, these image data are used in veterinary anatomy as educational tools, and for differentiating breeds [1,2,3,4]. Quantitative radiography allows not only subjectively assessment of the condition of the digital organ in particular individuals, but to learn about the changes that the phalanges undergo with age [5]. Radiological examination is crucial in revealing the source of orthopedic problems in horses [6,7]. In particular, a significant number of cases of lameness in thoracic limbs may be due to the non-optimal alignment of phalanges [8]. Various studies have been carried out to examine the position of phalanges relative to each other and the angular positions between them radiogrametrically [9,10,11]. In addition, X-ray images have been used to evaluate the general joint conformation of healthy animals, not just in suspicion of lameness [12]. Radiographic techniques can provide information regarding relationships between the hoof capsule conformation and the geometry of the distal phalanx [13,14]. Cohen et al. [15] attempted to determine prospectively the association between the abnormal radiographic findings and performance indicators in English Thoroughbred horses.

As stated above, the positions and conformations of phalanges are very important for clinical diagnosis. In this regard, it is thought that the reference information obtained from healthy animals will be valuable for future studies on this subject [16]. In addition, while collecting radiometric reference data on digital bones of the thoracic limbs of healthy animals, we tried to answer the following study questions:

Is there any difference in radiometric measurements of digital bones of the thoracic limbs of Arabian horses and Thoroughbred horses?

Can gender determination be made in radiometric measurements of digital bones of the thoracic limbs?

## 2. Materials and Methods

The study was conducted with 25 Arabian horses (7 females, 18 males) and 50 (15 females, 35 males) Thoroughbred horses aged between 2 and 8 years, brought to the Jockey Club of Turkey for control purposes. We used the unit of age as years. Horses that had no musculoskeletal or orthopedic problems before and actively participated in races and training were used in the study. Latero-medial radiological images of the digital bones of their thoracic limbs were acquired with Gierth X-ray (TR9030). The difference between the right–left manus was ignored, and only the images of the digital bones of the left thoracic limbs were used for analyses.

**Figure 1:**
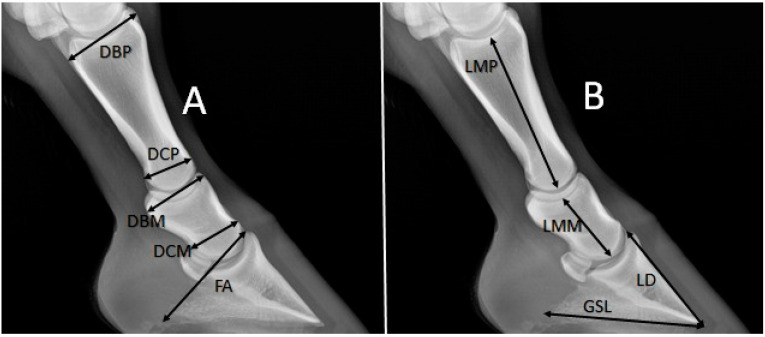
Measurement (4-year-old male Arabian horse). (**A**): Depth of the basis of the proximal phalanx (DBP), depth of the caput of the proximal phalanx (DCP), depth of the basis of middle phalanx (DBM), depth of the caput of the middle phalanx (DCM), length of the caudo-dorsal surface of the distal phalanx (FA). (**B**): Length of the middle of the proximal phalanx (LMP), length of the middle of the middle phalanx (LMM), length of the dorsal surface of the distal phalanx (LD), greatest solear length of the distal phalanx (GSL).

Three measurements of each of the proximal phalanx, middle phalanx, and distal phalanx were taken (Figure 1). Measurements were recorded using the Radiant DICOM Viewer (version 2020.2.2) software:

Proximal phalanx:

1. Depth of the basis (DBP), greatest depth.
2. Depth of the caput (DCP), greatest depth.
3. Length of the middle (LMP).
4. Index 1 (DBP × DCP/LMP).

Middle phalanx:

1. Depth of the basis (DBM), greatest depth.
2. Depth of the caput (DCM), greatest depth.
3. Length of the middle (LMM).
4. Index 1 (DBM × DCM/LMM).

Distal phalanx:

1. Length of the dorsal surface (LD), length of facies parietalis of the distal phalanx.
2. Greatest solear length (GSL), distance between the crena marginis solearis and the processus palmaris.
3. Length of the caudo-dorsal surface (FA), distance between the processus extensorius of the distal phalanx and the processus palmaris.
4. Index 3 (LD × GSL/FA).

SPSS (version 22) was used for statistical calculations. Differences between Arabian and Thoroughbred horses in terms of the morphometric features were evaluated separately for proximal phalanx, middle phalanx, and distal phalanx. Male and female samples of the two breeds were evaluated separately. In addition, measurement differences between the two horse breeds were analyzed regardless of gender. ANOVA was used for these analyses. Homogeneity of variances was examined, and *p* values were obtained. Discriminant function analysis was applied to reveal the differences between Arabian horses and Thoroughbred horses. Eigenvalue and Wilks’ lambda values were examined. A confusion table was used to evaluate the validity of DFA results. The correctly classified values and a discriminant distribution table were obtained with the Past (4.01) statistics program. Correlations between variables of the phalanges and between phalange traits and horse age were examined as well.

## 3. Results

The mean values of particular traits and standard deviation determined separately for the males and females of Arabian horses and Thoroughbred horses are presented in Table 1. The measurement points selected for Thoroughbred horses had no significant effect on the difference between sexes, as indicated by ANOVA results. The distribution of the samples between both breeds and sexes was homogeneous. Trait values in males were found to be higher for the proximal phalanx and middle phalanx. In Arabian horses, LMP (*p* < 0.05), LMM (*p* < 0.001), and LD (*p* < 0.05) were found to be significantly different between sexes. Values of all traits and indexes were found to be higher in the males of Arabian horses. According to the index results used, index 3 was a determinant between sexes only in Arabian horses (*p* < 0.05).

Table 2 shows the mean values, standard deviations, and statistical differences in phalangeal traits between Arabian and Thoroughbred horses regardless of sex. All traits and index values of the proximal phalanx and middle phalanx can be used in breed distinction. The difference in phalangeal traits was significant. The proximal phalanx and middle phalanx values were higher in Thoroughbred horses. The distal phalanx values were not statistically significantly different between the breeds. The GSL and FA values were found to be higher in Arabian horses, unlike other length measurements.

Results of the discriminant function analysis between Arabian and Thoroughbred horses are given in Table 3. Separate tests were performed for proximal phalanx, middle phalanx, and distal phalanx. Separate formulas were obtained for each bone. The highest canonical correlation value in the breed discrimination was obtained for the proximal phalanx. Multivariate discriminant function analysis score equations were as follow:

Proximal phalanx: (DBP × −16.584) + (DCP × −28.755) + (LMP × 6.686) + (Index 1 × 50.261) + 25.973.

Middle phalanx: (DBM × 0.931) + (DCM × 2.555) + (LMM × 2.187) + (Index 2 × −1.026) − 17.503.

Distal phalanx: (LD × −1.969) + (GSL × 1.706) + (FA × 0.454) + (Index 3 × 0.012) − 4.331.

For the proximal phalanx, the most distinctive feature in the discriminant function analysis was DCP (structure matrix: −0.801). In turn, LMM (structure matrix: 0.951) was the most distinguishing feature for the middle phalanx and FA (structure matrix: 0.666) for phalanx distalis. The eigenvalue value for the proximal phalanx was −0.909. It allowed correct classification of 89.33% of the samples used in the study (Table 4). The distinguishing features were the most tangible in the proximal phalanx; which was indicated by the highest percentage of the correctly classified bones. In the case of middle phalanx and distal phalanx, the correct classifications reached 77.33% and 54.67%, respectively. Traits of the distal phalanx were insignificant in the breed distinction. Wilks’ lambda value was very high (0.958).

**Figure 2:**
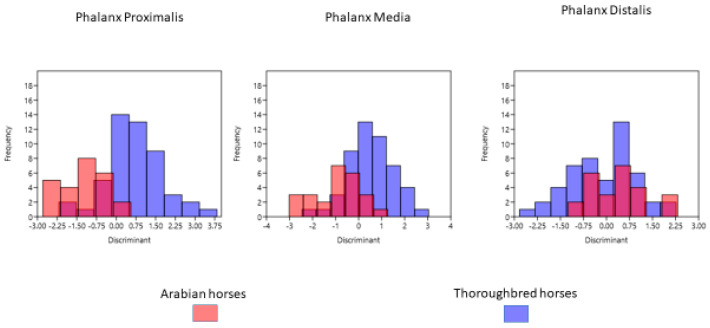
Distribution frequency of Arabian horses and Thoroughbred horses after discriminant analysis.

The distribution frequency of Arabian horses and Thoroughbred horses is shown in Figure 2. This figure demonstrates that the division into breeds based on the proximal phalanx data was more distinct. It can be seen that overlaps were more frequent in the distal phalanx. Almost none of the characteristics of the distal phalanx in Arabian horses differed from those of Thoroughbred horses.

Correlation values are presented in Table 5. There was usually a significant positive correlation between the individual measurement values. The length measurements of all three phalanges correlated strongly with the other parameters. The strongest correlation was found between the DCP and DBM.

## 4. Discussion

The phalanges of thoracic limbs of 75 horses were examined in the study. The LMP, LMM, and LD values were statistically different between males and females in Arabian horses, while no significant difference was found between males and females of Thoroughbred horses. Between the breeds, DCP was found to be more determinant for the proximal phalanx. In turn, LMM was the most distinguishing morphometric feature for the middle phalanx. It was observed that in Thoroughbred horses, most length measurement results were higher than those of Arabian horses (DBP, DCP, LMP, DBM, DCM, LMMLD, and FA). The reason for this is that Thoroughbred horses are generally taller than Arabian horses and this might be related to the length of individual sections of the limb skeleton [17]. The traits of the distal phalanx provide limited possibilities for breed differentiation.

Only radiogrametric measurements of digital bones of the left thoracic limbs of Arabian horse and Thoroughbred horse were used in this study. The difference between the right and left bones was ignored; only bone measurements were used to examine whether there was a sex and breed distinction. Previous studies have proved the lateralization of the skeleton of the manus. Alrtib et al. [15] compared right and left proximal phalanx measurements in their study using 10 Thoroughbred horses, five Standardbred horses and eight ponies. It was said that the medial length of proximal phalanx on the right side was larger than the left side. This difference was statistical. According to Kummer et al. [11], the left LMM is greater than the right and this difference is statistically significant. He reported that the left LMM before trimming was 4.6 ± 0.24 cm. In addition, he stated that the LMM correlates positively with the height at the withers. In contrast, Linford et al. [12] did not observe significant differences between the left and right phalanges in any radiographic determination. In our study, LMM was 3.9 ± 0.27 cm in Arabian horses and 4.3 ± 0.26 cm in Thoroughbred horses.

Measurements and 3D-modeling using imaging systems has become an alternative to traditional anatomical morphometry. In the study of Dos Reis et al. [2], it was stated that the 3D models successfully reflect the anatomical characteristics of bones and that measurements taken using these models approximate the actual bone measurements. In their study using 3D printing, the length of the proximal phalanx was 8.42 cm and the length of the middle phalanx was 4.48 cm. No information was given about the breed of horses used in this research, but the length of phalanx media was close to the respective value found in this study for Thoroughbred horses.

The mean value of the length of the proximal phalanx was 9.28 cm and the length of the middle phalanx media was 3.9 cm in Arabian horses, whereas in the Thoroughbred horses the respective values reached 9.89 cm and 4.3 cm. We examined whether there was a difference between the two breeds in radiogrametric measurements taken of phalangeal bones. Results of the proximal phalanx were found to be more distinctive than those of the other digital bones in breed differentiation. Dzierzęcka and Komosa [18] also revealed a statistically significant (*p* ≤ 0.01) difference in the greatest length of the proximal phalanx between warmblood horses and coldblood horses. They stated that the length of the proximal phalanx plays an important role in a certain morphological classification of animals.

In this study performed with Arabian horses and Thoroughbred horses, a correlation between the age of animal and osteometric features of phalanges was also evaluated. Analyses conducted demonstrated a positive correlation between the greatest solear length of the distal phalanx and age. Some studies can be found in literature addressing the effects of increasing age in horses on digital radiological measurements. Mullard et al. [19] proved that the ratio of the hoof distal phalanx distance to the length of the palmar aspect of the distal phalanx in horses decreases with increasing age, and that this correlation is statistically significant. In this study, the correlation of age with GSL was positive and only this value was statistically significant (R = 0.381). Several traits were negatively correlated with age, but these values were statistically insignificant. Anderson et al. [20] proved that there was no significant increase of length measurements in horses from age 2 to 3 years, suggesting that the growth rate either slowed or reached a plateau between ages 2 and 3 years. The lack of relationship of most analyzed parameters with age in our study is presumably due to the fact that most of the growth and development in the distal limbs of the horses ended at the age before the youngest of the measured horses. According to Sadek et al. [21], correlations between body measurements in Arabian horses vary a lot for both genders. Cruz et al. [22] examined the magnetic resonance images taken on these bones and stated that the exercise caused a positive change in the width parameters of the distal phalanx. In turn, Turek et al. [23] reported that different factors, including age, feeding, exercise and breed, might determine mechanical properties of the proximal phalanx in horses.

Osteometric measurements of phalanges were taken on equine bone remains obtained from excavations. Forsten [24] measured bones of horses in the Steinheim region. The length of the proximal phalanx found in this study was 9.13 ± 0.08 cm, and the length of the middle phalanx was 5.1 ± 0.14 cm. In another study, horses’ remains were examined in Üzüür Gyalan Tomb, and measurements of proximal phalanx and middle phalanx were taken of these bone remains. The greatest length of the proximal phalanx from this excavation was 8.47 cm and the greatest length of the phalanx media was 5.00 cm [25]. In the present study conducted with Arabian horses and Thoroughbred horses, reference measurement values were obtained radiogrametrically from contemporary samples. These reference data may suggest that in terms of taxonomy, they can be helpful in interpreting osteoarchaeological studies. These data are particularly comparable with bone remnant measurements obtained from excavations, and can provide information on animals of ancient periods. Although, when using these data, it should be kept in mind that there may be differences between measurements taken of bones and measurements taken via radiological imaging, as shown in the reference study [26].

The authors are aware that the lack of significance of differences between breeds and genders is partly due to the low abundance of research material. Another limitation is the lack of zoometric data, including the height at the withers, concerning the tested horses.

## 5. Conclusions

In this study, the statistical significance of radiogrametric measurements of the skeleton of the manus was examined in terms of sex and breed. Measurements of male individuals were generally greater. However, it can be concluded that the proximal phalanx and the middle phalanx manifest sexual dimorphism. The traits of the distal phalanx used in the study did not discriminate sex or breed. These data can serve as a reference material for radiogrametric studies in animals. The present study allowed obtaining reference radiogrametric measurements of the digits of the manus of Arabian horses and Thoroughbred horses.

## Figures and Tables

**Table 1:** Mean values of measurements, standard deviations, and *p* values determined between sexes of Arabian horses and Thoroughbred horses (ANOVA).

		Thoroughbred Horses				Arabian Horses			
Measurement	Sex	*n*	Mean (cm)	SD	*p* Value	*n*	Mean (cm)	SD	*p* Value
DBP	Female	15	4.72	0.25	0.84	7	4.30	0.14	0.06
	Male	35	4.74	0.30		18	4.46	0.20	
DCP	Female	15	2.99	0.13	0.05	7	2.73	0.10	0.07
	Male	35	3.09	0.19		18	2.83	0.13	
LMP	Female	15	9.82	0.38	0.44	7	8.99	0.39	0.03
	Male	35	9.93	0.47		18	9.39	0.37	
Index 1	Female	15	1.44	0.10	0.32	7	1.31	0.06	0.37
	Male	35	1.48	0.15		18	1.35	0.11	
DBM	Female	15	4.02	0.19	0.23	7	3.69	0.25	0.11
	Male	35	4.11	0.24		18	3.85	0.19	
DCM	Female	15	3.02	0.22	0.53	7	2.70	0.10	0.06
	Male	35	3.06	0.21		18	2.86	0.20	
LMM	Female	15	4.28	0.18	0.71	7	3.67	0.32	0.00
	Male	35	4.31	0.29		18	4.00	0.19	
Index 2	Female	15	2.85	0.31	0.36	7	2.73	0.17	0.75
	Male	35	2.93	0.26		18	2.76	0.28	
LD	Female	15	6.81	0.37	0.77	7	6.40	0.41	0.03
	Male	35	6.84	0.44		18	6.92	0.52	
GSL	Female	15	8.63	0.59	0.96	7	8.45	0.53	0.06
	Male	35	8.62	0.62		18	8.92	0.52	
FA	Female	15	6.28	0.36	0.33	7	6.19	0.48	0.38
	Male	35	6.16	0.43		18	6.37	0.43	
Index 3	Female	15	9.36	0.81	0.38	7	8.75	0.51	0.02
	Male	35	9.61	0.93		18	9.72	0.98	

**Table 2:** Mean values of measurements, standard deviations, and *p* values determined for Arabian horses and Thoroughbred horses (ANOVA).

Measurement	Breed	*n*	Mean (cm)	SD	*p* Value
DBP	Arabian	25	4.42	0.19	0.00
	Thoroughbred	50	4.73	0.28	
DCP	Arabian	25	2.80	0.13	0.00
	Thoroughbred	50	3.06	0.18	
LMP	Arabian	25	9.28	0.41	0.00
	Thoroughbred	50	9.89	0.45	
Index 1	Arabian	25	1.34	0.10	0.00
	Thoroughbred	50	1.47	0.14	
DBM	Arabian	25	3.81	0.22	0.00
	Thoroughbred	50	4.09	0.23	
DCM	Arabian	25	2.82	0.19	0.00
	Thoroughbred	50	3.05	0.21	
LMM	Arabian	25	3.90	0.27	0.00
	Thoroughbred	50	4.30	0.26	
Index 2	Arabian	25	2.75	0.25	0.03
	Thoroughbred	50	2.90	0.27	
LD	Arabian	25	6.78	0.54	0.63
	Thoroughbred	50	6.83	0.42	
GSL	Arabian	25	8.79	0.55	0.26
	Thoroughbred	50	8.63	0.60	
FA	Arabian	25	6.32	0.44	0.24
	Thoroughbred	50	6.19	0.41	
Index 3	Arabian	25	9.45	0.97	0.71
	Thoroughbred	50	9.53	0.90	

**Table 3:** Stepwise discriminant function analysis for Arabian horses and thoroughbred horses.

	V	UC	Constant	SM	WL	E	GC	CC
Proximal phalanx	DBP	−16.584	25.973	−0.614	0.524	−0.909	A: 1.330 T: −0.665	0.690
	DCP	−28.755		−0.801				
	LMP	6.686		−0.705				
	Index 1	50.261		−0.520				
Middle phalanx	DBM	0.931	−17.503	0.780	0.638	0.567	A: −1.051 T: 0.525	0.602
	DCP	2.555		0.721				
	LMM	2.187		0.951				
	Index 2	−1.026		0.353				
Distal phalanx	LD	−1.969	−4.331	−0.267	0.958	0.044	A: 294 T: −1.147	0.206
	GSL	1.706		0.632				
	FA	0.454		0.666				
	Index 3	0.012		−0.208				

V: variables, UC: unstandardized coefficient, SM: structure matrix, WL: Wilks’ lambda, E: eigenvalue, GC: group centroids, CC: canonical correlation. A: Arabian horses T: thoroughbred horses.

**Table 4:** Confusion matrix. Percentage of initial classifications that were correct, shown by breed.

		Arabian	Thoroughbred	
Proximal phalanx	Arabian	23	2	89.33%
	Thoroughbred	6	44	
Middle phalanx	Arabian	20	5	77.33%
	Thoroughbred	12	38	
Distal phalanx	Arabian	15	10	54.67%
	Thoroughbred	24	26	

Percentages within rows sum to 100%.

**Table 5:** Coefficients of correlation between measurements of digital bones and between these measurements and horse age.

	DBP	DCP	LMP	DBM	DCP	LMM	LD	GSL	FA
Age	−0.021	−0.020	0.076	−0.064	−0.121	−0.030	0.204	0.381 **	0.227
DBP		0.609 **	0.612 **	0.671 **	0.456 **	0.698 **	0.425 **	0.142	0.055
DCP			0.508 **	0.692 **	0.686 **	0.667 **	0.339 **	0.020	−0.043
LMP				0.491 **	0.355 **	0.691 **	0.280^*^	0.234 *	0.080
DBM					0.794 **	0.749 **	0.352 **	0.104	0.104
DCP						0.615 **	0.272 *	0.069	0.085
LMM							0.423 **	0.128	0.039
LD								0.559 **	0.299 **
GSL									0.760 **

** Correlation is significant at 0.01 level. * Correlation is significant at 0.05 level.

## Data Availability

Not applicable.
